# Spatially and angularly resolved spectroscopy for in situ estimation of particle size and concentration in particle suspensions

**DOI:** 10.1007/s00216-026-06696-8

**Published:** 2026-07-29

**Authors:** Carla Ferreira, Javier Cardona, Okpeafoh Agimelen, Christos Tachtatzis, Ivan Andonovic, Jan Sefcik, Yi-Chieh Chen

**Affiliations:** 1 CMAC, University of Strathclyde, Glasgow, G1 1RD UK; 2 Department of Chemical and Process Engineering, University of Strathclyde, Glasgow, UK; 3 Department of Electronic and Electrical Engineering, University of Strathclyde, Glasgow, UK

**Keywords:** Particle size distribution, Light scattering, In-line monitoring, Chord length distribution, Spatially and angularly resolved diffuse reflectance measurement (SAR-DRM)

## Abstract

Accurate in-line monitoring of particle size and solid concentration in turbid, multiphase systems remains a significant challenge in pharmaceutical manufacturing. Most particle measurement techniques are developed for off-line analysis, while recent in-line methods mainly rely on imaging and chord length distribution (CLD) measurement. Complementing these approaches, the spatially and angularly resolved diffuse reflectance measurement (SAR-DRM) system serves as a process analytical technology analyzer, capturing detailed, configuration-dependent spectral responses of particle suspensions. This study systematically evaluates SAR-DRM’s effectiveness in analyzing a wide range of particle diameters (≤90–800 µm) and concentrations (1–10 wt%) for polystyrene suspensions. The visible-NIR and NIR measurements from its multiple spatial-angular fiber configurations are further analyzed to assess their potential in complementing other in-line techniques, improving interpretability and analytical performance. The results show that combining specific SAR-DRM configurations through data augmentation markedly enhances model performance compared to individual configurations or co-adding methods, reducing error by over 50% in some cases. Moreover, data fusion of SAR-DRM with CLD measurements yields the most accurate models, particularly for mid-sized particles, decreasing the prediction error to 10–12 µm. The study highlights SAR-DRM as a promising process analytical technology analyzer for particulate process monitoring, paving the way for advanced hybrid modelling in pharmaceutical and chemical manufacturing. It also underscores the complementary sensing capabilities of SAR-DRM and CLD measurements, providing a robust multisensory platform for real-time, quantitative analysis in complex, high-turbidity systems.

**Graphical Abstract:**

**Figure:**
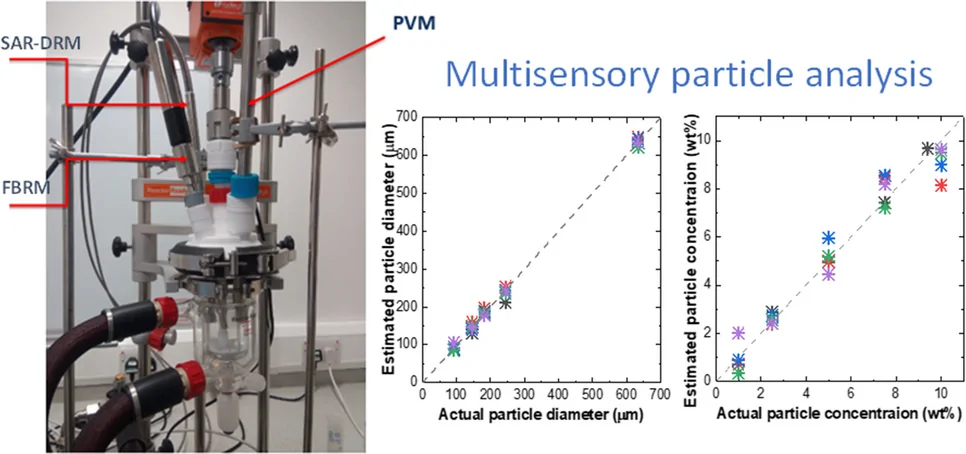

**Supplementary Information:**

The online version contains supplementary material available at https://doi.org/10.1007/s00216-026-06696-8.

## Introduction

The ability to monitor chemical attributes in chemical manufacturing processes in real time has been a major motivation for advancing process analytical technologies (PAT). However, many processes are multiphase and contain particulate substances, such as particles and droplets, and most PAT analyzers often overlook the physical attributes of these particulates. For example, particle size and shape are critical quality attributes for pharmaceutical ingredients because they directly influence downstream processing (e.g., isolation, formulation) and the performance of the finished product (e.g., uniformity, dissolution, bioavailability, stability) [1–4]. The particle attributes, and the changes in them, are of primary importance for processes such as crystallization, milling, and granulation. Various optical-based measurements (such as imaging, backscattering, and laser diffraction) or ultrasonic sensing technologies are developed to provide key attributes. However, controlling and monitoring the particle properties during the process remains a challenging field of research. Significant limitations in delivering real-time quantitative data are faced due to the speed of data processing and analysis, as well as the ill-posed nature of the inverse problems, where small changes in the measured signal lead to dramatic changes in the corresponding causes [5, 6].

An innovative spatially and angularly resolved diffuse reflectance measurement (SAR-DRM) system was recently developed for in-line monitoring in various chemical manufacturing applications [7]. The system acquires multi-wavelength (UV-visible-NIR) diffuse reflectance spectra from optical fibers with multi-angular and multi-spatial arrangements, utilizing wavelength-dependent optical properties—namely, scattering and absorption coefficients—to evaluate the chemical composition and particle attributes in suspensions. The measurement principle of SAR-DRM differs from many in-line particle analysis technologies, such as chord length distribution (CLD) measurement or particle imaging, which typically measure the optical response of a single particle (single scattering) at one time using a single-wavelength light source. Current in-line particle analysis technologies often offer an indication of particle size and shape rather than a precise measurement, such as solid concentration in a suspension. Additionally, higher solid concentrations increase the complexity of the acquired signals or images [8]. In highly turbid media, photons can undergo multiple light-scattering events before reaching a detector. Since light scattering is a non-linear process in which light’s direction changes after each event, multiple scattering can degrade the captured information and lead to misinterpretation of signals. This phenomenon imposes significant limitations on many in-line particle analysis techniques, which were developed assuming single scattering.

SAR-DRM captures the diffusely reflected signal at multiple wavelengths after multiple light scattering processes [9–11]. The technique is advanced from the diffuse reflectance measurement (DRM) technique, which analyzes the chemical composition of solid or turbid materials in the near-infrared region. For in-line applications, DRM typically employs optical probes designed to illuminate one or more specific locations; multiple detecting fibers are often used to collect and combine the optical signals into a single spectrum, providing an “average” overview of the material properties within the interrogated volume. However, this approach cannot resolve signals arising from local heterogeneity in physical and chemical composition. In SAR-DRM, the source and detecting fibers are positioned at specific spatial and angular relationships to capture signals from different light paths through the sample. Studies that utilize spatially resolved reflectance measurements have been reported to determine the optical scattering and absorption properties of heterogeneous samples [12–15]. These studies make use of the rich information contained in multi-wavelength spectra collected from different optical paths, enabling the extraction of detailed information about particle attributes and system behavior for a more comprehensive assessment.

Although SAR-DRM spectra can be inverted to determine bulk optical properties [7] and subsequently estimate particle size distributions (PSDs) [16], the inversion methods involve using Mie theory–based calculations, which are valid when the measurement wavelength is much greater than the particle size, such as when inverting visible-near infrared spectra for particles on the nanometer scale. The applicability and performance of extending this inversion method to micron-to-millimeter-sized suspensions remain uncertain. Furthermore, these methods require complex mathematical operations and intensive computational calculations, making them currently unsuitable for real-time applications. An alternative approach for analyzing SAR-DRM signals in particulate systems involves multivariate analysis (MVA). A study employing MVA on SAR-DRM spectra reports that this technique outperforms common reflectance measurements for highly turbid suspensions with particles smaller than 1 µm [14]. The study also investigates the effect of spatial distances on MVA and explores methods for combining spectra from different configurations to further improve model performance. However, for samples containing particles in the micron-to-millimeter range, the performance of SAR-DRM is not yet fully understood. This size range is particularly important for monitoring and controlling APIs used in the production of oral dosage forms such as tablets and capsules. Although the potential of SAR-DRM in analyzing micron-to-millimeter-sized particle suspensions is briefly discussed in a feasibility study [17], a thorough assessment of SAR-DRM’s performance remains necessary.

This work investigates the applicability of SAR-DRM for estimating particle size and concentration in suspensions with particles ranging from micron to millimeter scale. As this is the first detailed study utilizing the SAR-DRM system to analyze particles within this size range, the results are divided into three sections. First, the sensitivity, accuracy, and capabilities of SAR-DRM in both the visible and near-infrared spectral regions for distinguishing differences in particle size and concentration are evaluated. Next, the performance of multivariate regression models developed with SAR-DRM’s multiple spatial-angular fiber configurations for estimating these parameters is examined. This includes applying the analysis to spectra from individual spatial and angular configurations, followed by an evaluation of the improvements gained when using models constructed on multiple configurations. Finally, the potential for SAR-DRM to complement another in-line technique—CLD measurement—based on the single scattering effect is discussed.

## Materials and methodology

### Materials and experimental setup

Polystyrene (PS) particles suspended in water served as the model system. Since the particles were synthesized via suspension polymerization, a wide range of sizes and shapes was observed, with shapes gradually shifting from ellipsoids to spheres as their size increased [8, 12]. Five particle diameter groups, ranging from < 90 to 800 µm, were established after sieving. Off-line laser diffraction analysis (Mastersizer 3000, Malvern Instruments Limited, UK) was used as the reference method for particle diameter. Particle size of the sample, expressed as equivalent spherical diameter, is inverted using the built-in function in the laser diffraction analyzer. The particle size distributions (PSD) of all particle size groups are shown in SFigure 1 of the Electronic Supplementary Material. For the multivariate analysis in this study, the cumulative percentile values of 10, 50, and 90 (D10, D50, and D90, respectively) for each PSD, summarized in Table 1, are used as the dependent variables. Table 1 also includes the size ranges and the corresponding nomenclature.

**Table 1:** PS size groups used and the corresponding D10, D50, and D90 from laser diffraction, in this study

Diameter range	Sample name	D10	D50	D90
< 90 µm	PS0-90	48	92	148
125–180 µm	PS125-180	109	146	206
180–250 µm	PS180-250	112	182	253
250–355 µm	PS250-355	227	244	295
630–800 µm	PS630-800	486	634	732

The SAR-DRM and CLD measurements were taken simultaneously using the setup reported in Ref. [8]. In brief, particles were suspended in deionized water within a 250-ml glass jacketed tank reactor (Radleys, UK) and agitated at 500 rpm by a PTFE turbine stirrer during the measurement to avoid particle sedimentation. The setup (including probe positions and direction) was optimized to reduce interference between different optical measurement techniques. The CLD probe (FBRM G400 system, Mettler-Toledo, UK) was set at an inclination of 30°, while the SAR-DRM probe was positioned vertically in the vessel, as shown in SFigure 2 (a) in the Electronic Supplementary Material. To prevent ambient light from affecting the SAR-DRM spectra, the reactor vessel was covered with a dark cloth. Five particle concentrations with solid loadings of 1.0, 2.5, 5.0, 7.5, and 10 wt% were prepared, resulting in a total of 25 samples with varying sizes and concentrations.

### Diffuse reflectance measurement using SAR-DRM

The SAR-DRM system measured diffuse reflectance caused by light scattering of the PS particles. A detailed description of the SAR-DRM system was provided in Ref. [7]. In this study, the light source was replaced with a custom-made unit that illuminates with a 50-W tungsten-halogen lamp powered by 12 V. The lamp was positioned at the focal point of an ellipsoidal reflector (Part no. 90–969, Edmund Optics, UK), and the lamp housing couples the light into the source fibers in a ferrule, which was placed at the other focal point of the reflector to maximize the optical throughput. The setup for the other components of the SAR-DRM system remains unchanged.

The SAR-DRM probe emits light at incident angles of 0°, 30°, and 45° and collects diffuse reflectance using 16 fibers positioned at 0.3, 0.6, 0.9, and 1.2 mm from the source fibers, as illustrated in SFigure 2(b) in the Electronic Supplementary Material. The reflectance signal was captured with two spectrometers in the UV-vis-NIR spectral region (USB4000 and NIRQuest 2.2–512, Ocean Optics). In this study, the vis-NIR and NIR spectra cover the ranges of 500–900 nm and 1000–1850 nm, respectively. These wavelength ranges were chosen to avoid regions where the spectrometers have low quantum efficiency. The vis-NIR and NIR spectra were obtained using integration times of 30 ms and 2 s, respectively, and averaged over 25 and 5 spectra. The longer integration time for the NIR spectra accounts for the lower quantum efficiency of the InGaAs detector in the NIR spectrometer. For the vis-NIR spectra, interference from the chord length measurement via the laser light at 785 nm was detected, despite spacing the probes apart. This interference was mitigated by subtracting the dark spectra from the sample signal. The signal processing protocol for the SAR-DRM spectra follows previously described methods [5]. All SAR-DRM datasets underwent signal smoothing using a least-squares second-order polynomial with a 15-point smoothing window.

### Multi-block datasets

The SAR-DRM measurement produces 24 spectra for each sample, corresponding to 12 source-detecting fiber configurations for each spectral range. Table 2 summarizes the nomenclature of these 12 configurations. Each vis-NIR and NIR spectrum contains 3648 and 512 variables, respectively. Additionally, the final part of this study involves a dataset from square-weighted chord length measurements from Ref. [6], with each chord length distribution (CLD) comprising 100 variables. To ensure a consistent number of variables across datasets and avoid any impact on the MVA model’s performance due to variable count, data interpolation was performed using MATLAB’s “interp1” function to standardize all datasets to 350 variables.

**Table 2:** Nomenclature of the difference angular and spatial SAR-DRM configurations between the source and detecting fibers

Source-detecting fiber distance (mm)	Incident angle (°)		
	0	30	60
0.3	L1-D1	L2-D1	L3-D1
0.6	L1-D2	L2-D2	L3-D2
0.9	L1-D3	L2-D3	L3-D3
1.2	L1-D4	L2-D4	L3-D4

The 12 configurations in the SAR-DRM dataset for each spectral range can be regarded as 12 separate data blocks. Multiple data blocks can be combined to create a single data block using data fusion methods. Two methods, co-adding and augmentation, were used to combine the SAR-DRM data blocks from the same spectral range [7]. For combining datasets from different measurement techniques or spectral regions, only data augmentation was applied.

### Multivariate regression analysis

Partial least squares regression (PLSR) analysis was performed on the SAR-DRM and chord length measurements to build calibration regression models for polystyrene particle diameters (D10, D50, and D90) and concentration. Given the sample size and available conditions, leave-one-out cross-validation was employed. A logarithmic transformation was applied to the reflected signal recorded by SAR-DRM, similar to converting reflectance to absorbance, to linearize the SAR-DRM spectra. Both vis-NIR and NIR datasets were pre-processed using five common empirical methods: multiplicative scatter correction (MSC), extended MSC (EMSC), standard normal variate (SNV), and inverted scatter correction (ISC) for the SAR-DRM datasets. Mean centering was applied to all datasets. For the CLD dataset, only SNV and log transformation were used, as recommended in the previous study [8].

For each PLSR model, the number of latent variables (LVs) was selected based on the analysis of the root mean square error of cross-validation (RMSECV) curve against the number of LVs. The LV value that produced the lowest RMSECV was chosen if this represented a clear minimum. In cases where the RMSECV curve levels off after a significant initial drop, a lower number of LVs was selected to prevent including non-informative LVs in the model. When RMSECV decreases monotonically with an almost flat profile, a model with no fewer than 6 LVs was chosen. The minimum of 6 LVs was determined based on the number of system components that vary (particle size, particle size distribution, concentration of particles and water, different amounts of spherical and ellipsoidal particles present in the particle size group).

## Results and discussion

### Effect of particle size on SAR-DRM spectra

**Fig. 1:**
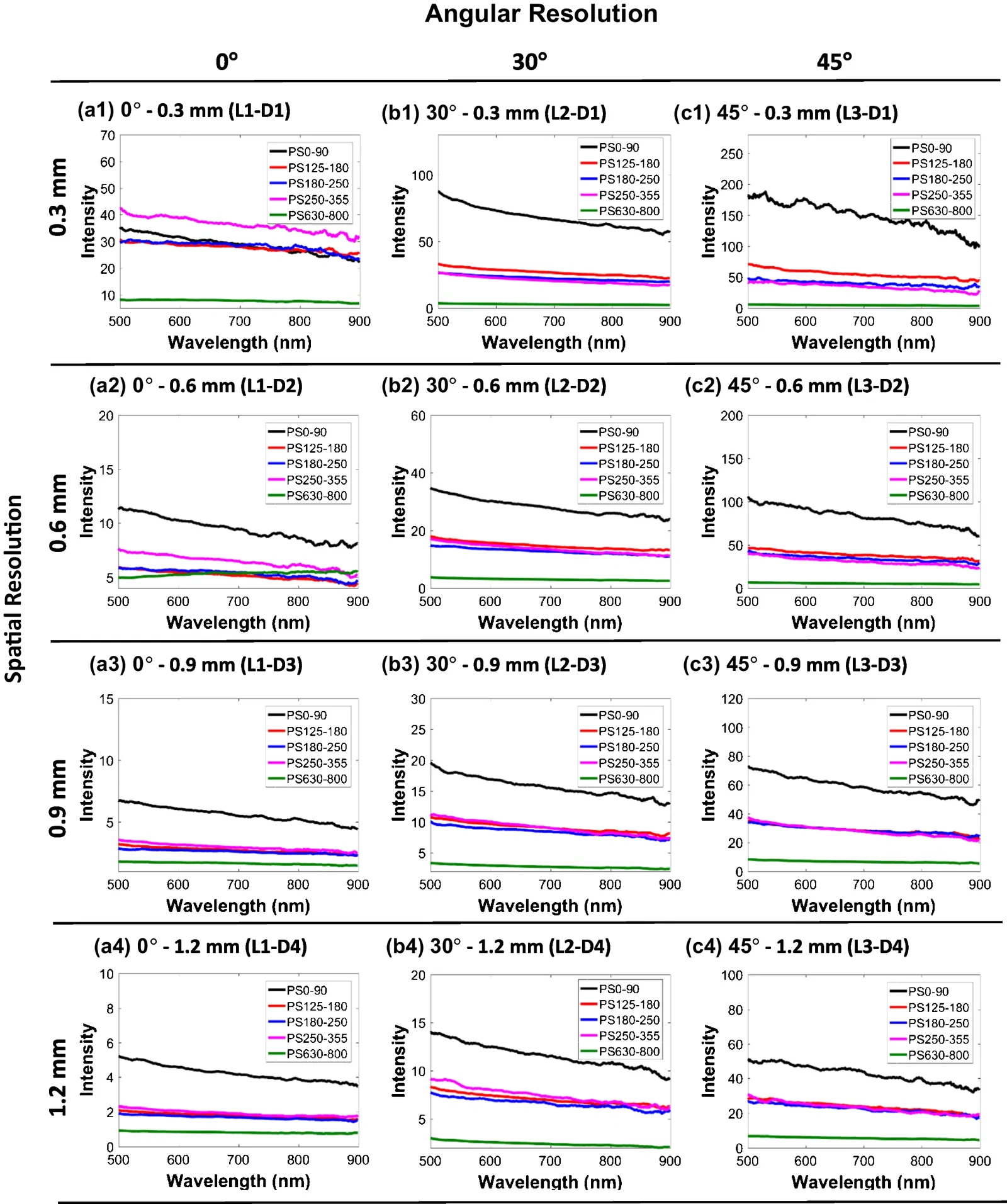
Effect of particle diameter on SAR-DRM spectra in the vis-NIR region using **a**–**c** 0, 30, and 45° incident light and **1**–**4** 0.3–1.2 mm spatial distances. 10 wt% particle concentration is used for all samples

The SAR-DRM system offers a total of 12 spatial and angular configurations between the source and detecting fibers for each wavelength region. Figure 1 (and SFigure 3 of the Electronic Supplementary Material) presents an example of SAR-DRM spectra from 10 wt% suspensions for all particle size groups for the vis-NIR (and NIR) regions. Overall, a decrease in the SAR-DRM intensity with increasing spatial distance from 0.3 to 1.2 mm can be observed. This is due to an increase in the optical path length with greater spatial distance, leading to stronger absorption and scattering of the incident light. Since the absorption of the sample (water and polystyrene) in the vis-NIR wavelength range is negligible, all SAR-DRM spectra in Fig. 1 show a smooth, featureless decrease in reflected intensity as wavelength increases. This trend can be attributed to a reduction in the scattering coefficient of the PS particles [18].

The spectra from PS0-90 show the strongest reflectance intensity for most source-detecting fiber configurations, except for L1-D1, which is due to the lower optical throughput caused by slight misalignment of the multiplexer. Increasing spatial distances results in increased similarity in the SAR-DRM spectra, regardless of the incident angle used. For example, the spectra collected at distances of 0.9 and 1.2 mm for PS125-180, 180–250, and 250–355 exhibit similar spectral intensities and are almost visually indistinguishable. In general, samples containing the largest particle size group, PS630-800, result in the lowest intensity. This can be attributed to the low particle number density for this particle size group. Large particles typically exhibit strong scattering properties; however, the bulk scattering property of a sample is determined by the multiplicative combination of the scattering coefficient of a single particle and the particle number density [19]. For a given particle concentration, the particle number density decreases as the particle diameter (and volume) increases. In this case, the decrease in the impact of particle number density on bulk scattering outweighs the increase due to particle diameter, resulting in the lowest reflectance signal observed on PS630-800. Similar findings are seen in the diffuse reflectance spectra from the NIR region, SFigure 3, in most cases.

Interestingly, by changing the angle of the incident light, Fig. 1b1–b4 and c1–c4 show noticeable differences between various particle size groups. Additionally, using angular incident light significantly boosts the reflected intensity across all sample conditions. This is probably because the optical path length is shortened by the angular incident light for the same source-detector distance. Compared to those with normal (0°) incident light, the path of angular incidence light within a sample is shallower and encounters fewer scattering events before exiting. By employing multiple spatial and angular arrangements, SAR-DRM measures the sample at different depths, offering sensitive and detailed reflectance data that can be used to derive the physical and chemical properties of the samples studied. These benefits are also seen in the NIR region, as demonstrated in SFigure 3 in the Electronic Supplementary Material. Similar to Fig. 1, the reflected intensity increases as the incident light angle shifts from 0 to 45°, with measurements at a normal (0°) incident angle being most effective for distinguishing particle size groups. This may be because normal incident light can penetrate deeper into the sample (for the same spatial distance), making it more affected by absorption and scattering, and more sensitive to the essential physical and chemical properties of the samples.

Analyzing the effect of particle size on SAR-DRM spectra is complex. The SAR-DRM spectra contain the combined scattering effect from multiple particles. The magnitude and direction of scattered light from a particle depend on its characteristics, such as size and shape, as well as the wavelength of the incident light. The analysis becomes even more complicated if the system’s absorption is significant, as is the case with the NIR spectra [20, 21]. A previous study on SAR-DRM spectra indicates that the mean particle size and concentration can be inferred from the spectra using a physics-based model [7]. However, given the size range and mixed shapes of the PS samples in this study, the physical model underlying the inversion method is not suitable, as it assumes spherical particles. Therefore, an empirical analysis of the vis-NIR spectra was conducted to evaluate SAR-DRM performance in distinguishing sample conditions by linearly fitting the spectra to derive their slope, a common spectral feature related to scattering.

**Fig. 2:**
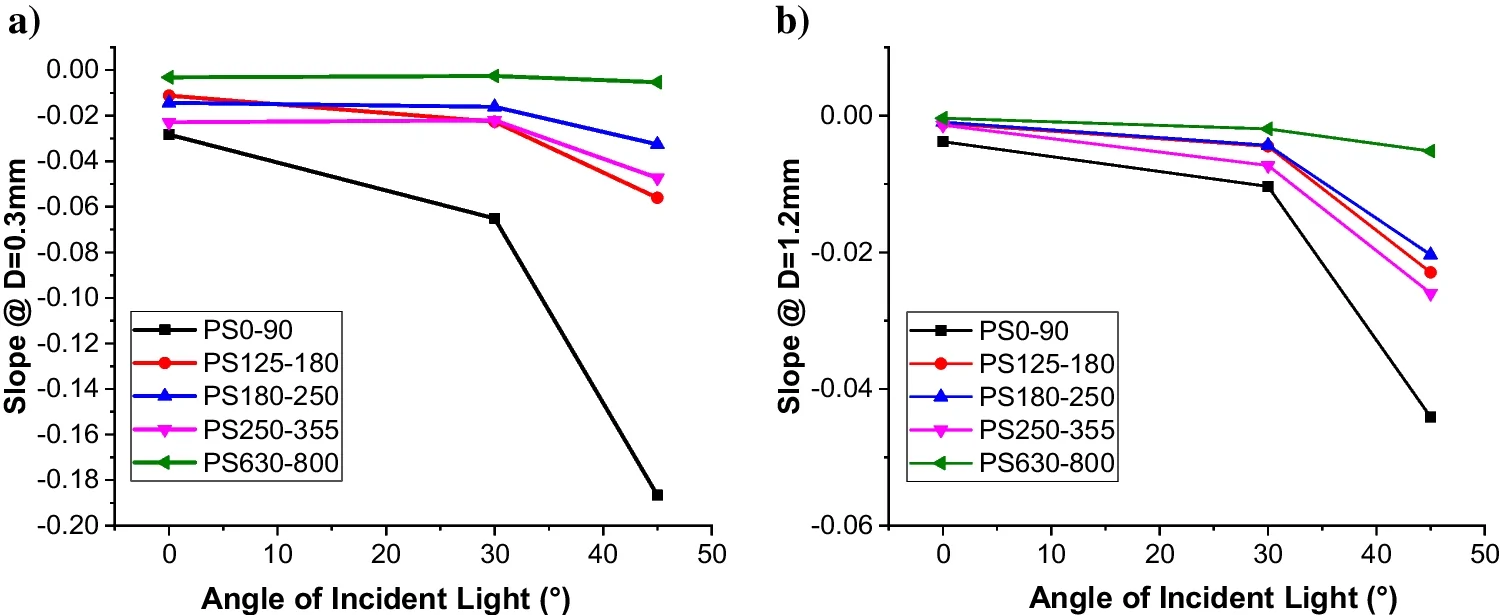
Slopes of SAR-DRM spectral baseline for samples of 10 wt% particle concentration using all angular incident angles and spatial distances of **a** 0.3 mm and **b** 1.2 mm

Figure 2 summarizes the fitted slope of spectra shown in Fig. 1a1–c1 and a4–c4. The slope generally decreases as particle size increases, with a few exceptions for spectra collected from a distance of 0.3 mm. A larger difference in the slope between the size groups is observed when using an angular incidence light source. The PS particles examined here are known to vary in shape distribution, with nearly 50% of the PS0-90 particles being ellipsoids [8]. Since the scattering properties of particles also depend on shape, the degree of shape uniformity can influence the SAR-DRM spectra, especially at short spatial distances. Given the particle size groups studied, it is likely that the SAR-DRM spectra reflect only a few scattering events caused by highly directional scattering from the diverse mixture of spherical and ellipsoidal particles in random orientations. This observation presents an interesting opportunity for future research on using SAR-DRM to estimate particle shape and shape distribution.

Overall, SAR-DRM exhibits a more distinct difference between particle size groups than either unweighted or volume-weighted CLD reported earlier [8]. The findings indicate that the spectral response under various combinations of spatial and angular arrangements can be further exploited to improve particle size analysis with higher sensitivity. Since scattering and absorption properties depend on the wavelength used, the observations in Fig. 1 and SFigure 3 emphasize the advantages of employing spectral measurements to capture wavelength-dependent optical responses.

### Effect of particle concentration on SAR-DRM spectra

**Fig. 3:**
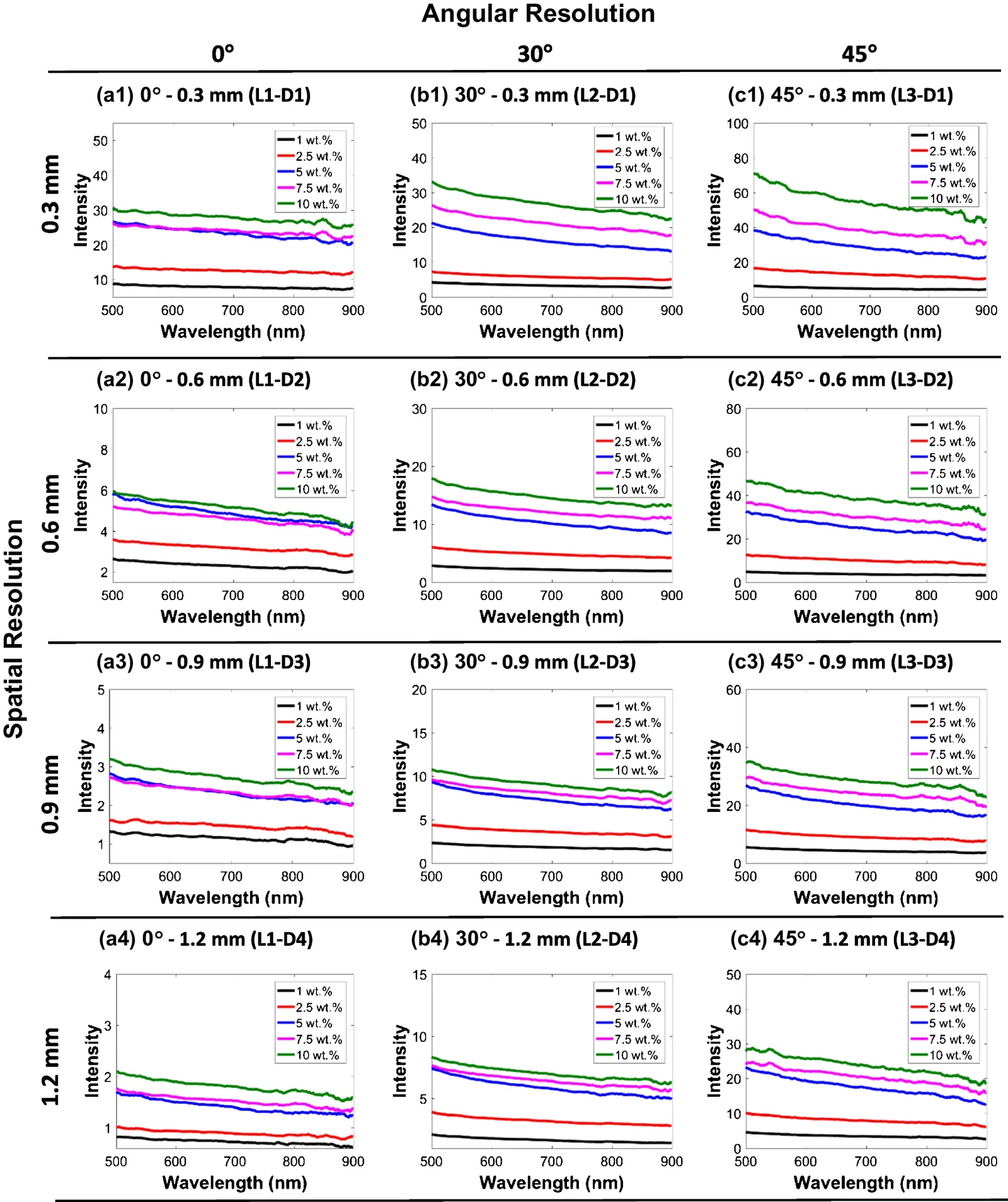
Effect of particle concentration of PS125-180 in the vis-NIR region using **a**–**c** 0, 30, and 45° incident light and **1**–**4** 0.3–1.2 mm spatial distances, respectively

A similar analysis is conducted to examine the effect of particle concentration on SAR-DRM spectra. An example of the changes observed with PS125-180 is shown in Fig. 3. Overall, increasing particle concentration leads to a consistent rise in the reflected light’s intensity. However, the change in intensity is non-linear and depends on the angular and spatial measurement configurations. For instance, SAR-DRM spectra show smaller intensity differences between various concentrations when an incident angle of 0° is used. As the spatial distance increases, this difference diminishes, as seen in Fig. 3b1–b4. Employing angular incident light results in more notable spectral separation between concentrations and a gradual decrease in intensity as the spatial distance grows.

A similar spectral response to Fig. 3 is also observed in the SAR-DRM NIR spectra, as shown in SFigure 4 in the Electronic Supplementary Material, and across other particle size groups. The interesting finding regarding the effect of incident angle highlights the importance of spatial and angular configurations in obtaining distinguishable information related to sample conditions. It also indicates the need for further analysis to exploit the rich information from the 12 configurations in this study, aiming to improve the understanding of light scattering properties in a turbid system.

**Fig. 4:**
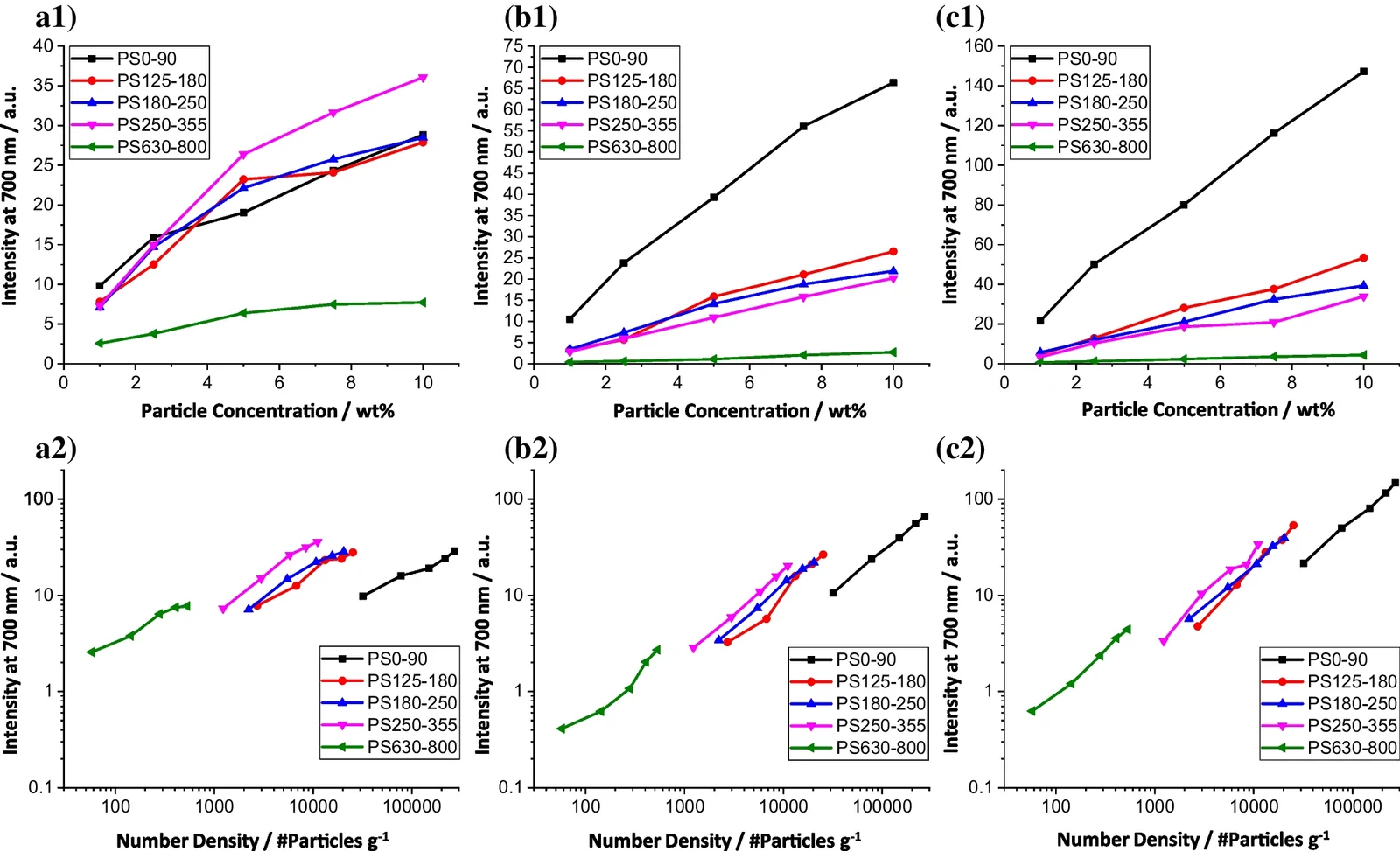
Particle concentration effect when using **a1** 0, **b1** 30, and **c1** 45 light source and a spatial distance of 0.3 mm. **a2**–**c2** show the corresponding number density for **a1**–**c1**

To quantify and compare the changes in SAR-DRM intensity with concentrations across different particle size groups, the intensity at a wavelength of 700 nm was chosen for further analysis. Using the smallest spatial distance as an example, Fig. 4a1–c1 shows that the configuration with angular incident light results in a greater intensity difference between various particle size groups. Increasing the concentration also enhances the differentiation among particle size groups. For example, Fig. 4b1 and c1 reveal a more distinct separation between PS125-180, PS180-250, and PS250-355 at concentrations above 2.5 wt%. This contrasts with the imaging and chord length analysis, where these techniques begin to show limitations as concentration increases.

Figure 4a2–c2 further illustrate the changes in SAR-DRM intensity with particle number density. However, unlike the in-line imaging and chord length measurements previously reported, the relationship between SAR-DRM intensity and particle number density is not straightforward. The SAR-DRM spectra are affected by confounding absorption and scattering effects, which result from multiple scattering, altering the optical path length of light travelling the sample. Fully describing this phenomenon would require developing a physical model based on light propagation theory to invert the spectra, as demonstrated by studies on suspensions of particles smaller than a micron [16–18]. However, since the particle size groups examined here are comparable to or larger than the wavelength used in measurements, the physics-based theory is not applicable. Moreover, relying on a single wavelength for particle information can be misleading. For example, analyzing the spatial distances from 0.6 to 1.2 mm yields less clear separation between particle size groups when using the intensity at 700 nm. It would be more reliable and robust to include multiple or a range of wavelengths to produce a more accurate analysis. Consequently, multivariate analysis (MVA) was carried out on the SAR-DRM spectra. By considering the spectral response collectively, this approach not only aids in understanding how best to utilize the detailed SAR-DRM spectral data but also evaluates whether SAR-DRM can complement other in-line analytical techniques.

### Effect of using one or multiple SAR-DRM configurations in estimating size and concentration of particles

The results shown above demonstrate a systematic response in the SAR-DRM spectra to the particle size and concentration of micron-sized particle suspensions. This section applies partial least squares regression (PLSR) analysis to the SAR-DRM spectra to explore the possibility of estimating these key sample features. In addition to modelling the 24 individual data blocks, which correspond to the visible and NIR spectra obtained from the 12 configurations, the data blocks can be combined to merge the captured information. However, this would create a large number of permutation combinations for combining different data blocks, requiring a systematic approach to assess the effectiveness of the combined dataset. The following steps were taken to evaluate the PLSR model performance in estimating particle size and concentration from various data blocks: (1) analyze the 24 individual data blocks; (2) analyze data blocks formed by combining all spectra from the 12 configurations; and (3) analyze data blocks formed by combining the two best-performing configurations for each illumination angle. The data block examined in Step 2 simulates the measurement setup typically provided by total diffuse reflectance measurements, which gather all reflected signals from a sample, regardless of the spatial and angular relationships to the incident light. Step 3 investigates the potential to enhance PLSR model performance by using the data blocks that contain more extractable information, as identified in Step 1. Data augmentation and co-adding methods are applied only to data blocks analyzed in Steps 2 and 3. The PLSR models are compared based on the number of LVs used, RMSECV, and the coefficient of determination (*R*^2^), after selecting models with an appropriate number of LVs for each data block, as described in the “Materials and methodology.”

In Step 1, the PLSR models for the 12 individual configurations are built using D10, D50, and D90 of the PSD as the dependent variables. Results of the model performance are summarized in Table 3 for those using D50 and STable 1 for D10 and D90. Also shown in Table 3 are the models for particle size and concentration obtained for the same individual configurations and those from Step 2 (the last two configurations). Table 3 and STable 1 show that the models built on individual fiber configurations produce a wide range of performance. For example, the RMSECV for estimating D50 ranges between 32 and 144 µm, and 1.2 and 2.9% for particle concentration. No incident angle or spatial distance can consistently produce spectra that lead to good model performance. Furthermore, the relative performance across the 12 configurations for the particle size models is consistent, regardless of the cumulative percentile values used. For example, using 0° incident light (L1), the vis-NIR spectra at spatial distances of 0.3 and 0.6 mm (D1 and D2) result in better model performance for estimating D10, D50, and D90.

**Table 3:**
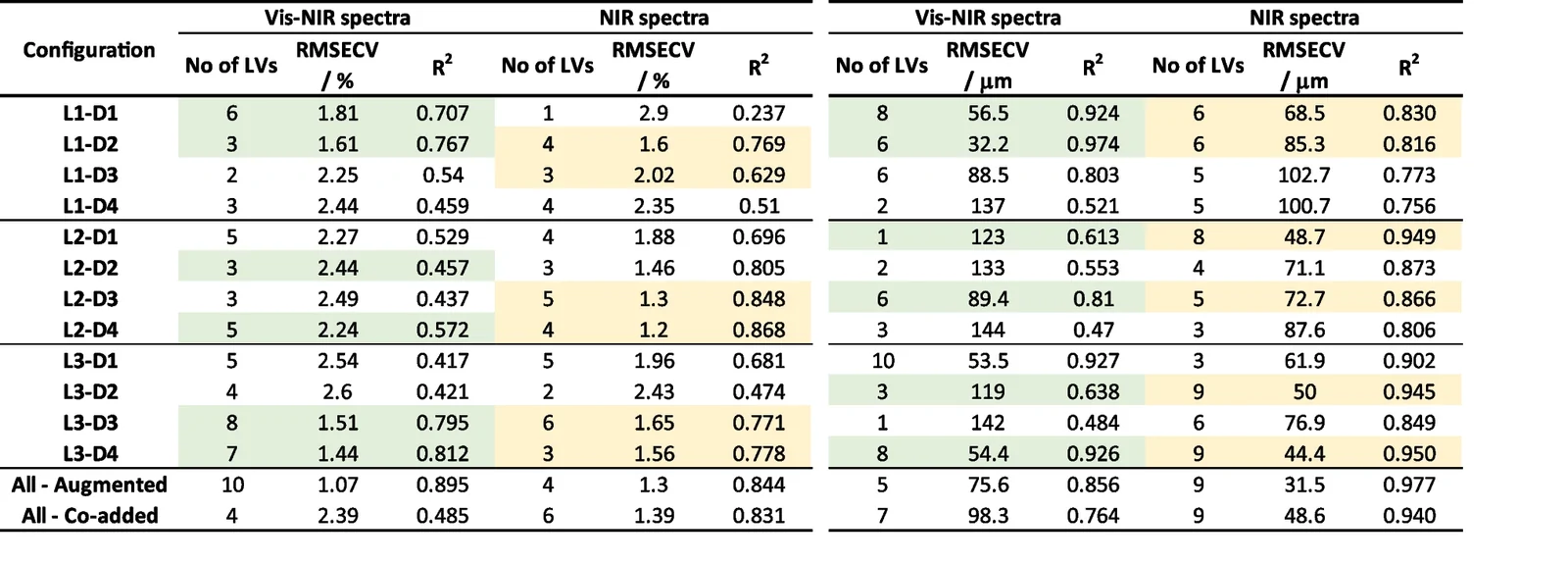
Summary of PLSR models for all available vis-NIR and NIR measurement configurations investigated in Steps 1 and 2 of the analysis. The two best-performing configurations for each incident angle are highlighted in colors

The findings from Step 1 can be attributed to multiple factors affecting the quality and information captured. The light scattering effect is a dominant factor in returning the reflected signal. However, the non-linear wavelength and angular dependence of the scattering effect, combined with the absorption properties of the particles and water, complicate the information obtained, as discussed in “Effect of particle size on SAR-DRM spectra” and “Effect of particle concentration on SAR-DRM spectra.” The spectral quality, such as the signal-to-noise ratio, is another factor that influences the model’s performance. A larger spatial distance results in increased absorption, especially in the NIR region, and reduces signal throughput; this can hinder the model’s ability to distinguish the signal from noise, thereby affecting its performance. Conversely, considering the sample conditions investigated, some of the spectra from shorter spatial distances may correspond to only a few scattering events, posing challenges in differentiating between samples with similar conditions, such as PS125-180 and PS180-250. Similar observations across models built with different cumulative percentile values can be attributed to systematic changes in the PSD, as shown in SFigure 1. The clear and systematic shift in PSD suggests that D50, as the most commonly used characteristic for particle population, would be representative for the further analysis steps in this study.

Results in Table 3 also reveal an interesting finding from Step 2. The last two configurations in Table 3 correspond to the combination of the SAR-DRM spectra from the 12 available configurations using the augmentation and co-adding approaches, respectively. In particular, the co-added configuration mimics the signal process commonly employed by probe-based spectroscopy systems. Compared to the results from Step 1, the models from augmented data show a notable improvement in estimating particle size and concentration. The co-adding of all available fiber configurations yields similar enhancements for models built on the NIR spectra. While co-adding may be regarded as a method to improve the signal-to-noise ratio of the obtained spectra [7], mixing responses from different optical pathways could also average out or cancel the sensitivity of the measurements, thereby reducing the ability to distinguish between samples under different conditions. Conversely, the augmentation approach preserves the distinct responses captured by each individual configuration, providing clear information for the regression analysis.

**Fig. 5:**
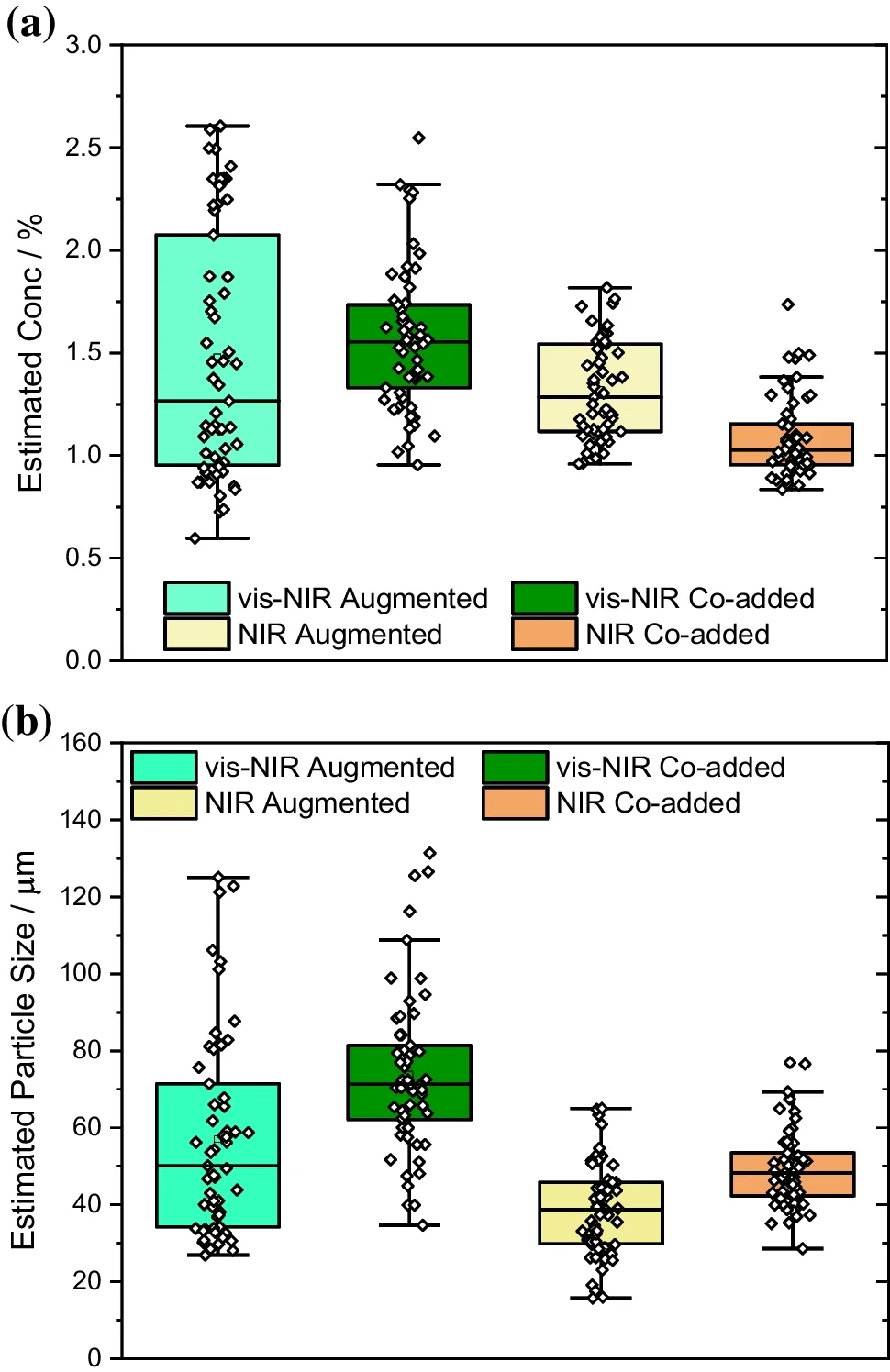
Comparison of the RMSECV on estimating **a** particle concentration and **b** particle diameter from all models built in Step 3. The open symbol corresponds to the RMSECV of the 57 permutation combinations analyzed

The drawback of the augmentation approach is the increased size of the dataset and the complexity of the PLSR model, which can subsequently raise the computing time needed for model construction and application. Step 3 is therefore undertaken to reduce the complexity of the PLSR model while maintaining or even enhancing its performance. Table 3 highlights 6 data blocks from the two best-performing fiber configurations for each incident angle. The 6 data blocks can generate 57 permutation combinations to form a combined dataset. Using the same principle to select the appropriate latent variable for each combination, the distributions of the RMSECV for the 57 models are analyzed and compared in Fig. 5.

Several interesting observations can be identified from the results in Fig. 5. Firstly, compared to the results from the NIR datasets, the models built on the vis-NIR datasets display a wider range of RMSECV values, regardless of the data combination method used. This may be caused by the combination of the featureless nature of the vis-NIR spectra and the larger intensity differences in these spectra at various spatial distances, leading to greater variation in the PLSR models when distinguishing spectral changes based on sample conditions. Additionally, co-added datasets produce a narrower RMSECV range across different data permutation combinations, as shown by the upper and lower quartiles of the box plot. This can be explained by the process of combining and removing spectral differences across configurations through co-adding, resulting in models that outperform those built on augmented datasets. Compared to the co-adding approach, models built on the augmented dataset generally exhibit lower mean and median RMSECVs, as well as a lower RMSECV for the best model. The only exception is the models for particle concentration using NIR spectra, where the co-added NIR datasets show better overall performance and the lowest RMSECV among the 57 models evaluated. The analysis in Fig. 5 indicates that using data augmentation to combine spectra from different configurations is advantageous.

A summary of the best-performing models for each spectral range is presented in Table 4, including the best model from the chord length measurement for comparison and the discussion in the next section. Also shown in Table 4 is the second-best model for estimating particle concentration using augmentation. This model uses the same fiber configuration as the overall best model for estimating concentration from NIR spectra. This additional information will be used in the next section’s discussion. The same permutation combinations of configurations are also analyzed by adding spectral pre-processing to the dataset to explore potential improvements. However, among the pre-processing methods tested (SNV, MSC, EMSC, and ISC), none of the processed datasets performed better than the models in Table 4. The RMSECV curves, along with the prediction and residual plots for all models in Table 4, are summarized in SFigures 5–7 in the Electronic Supplementary Material.

**Table 4:**
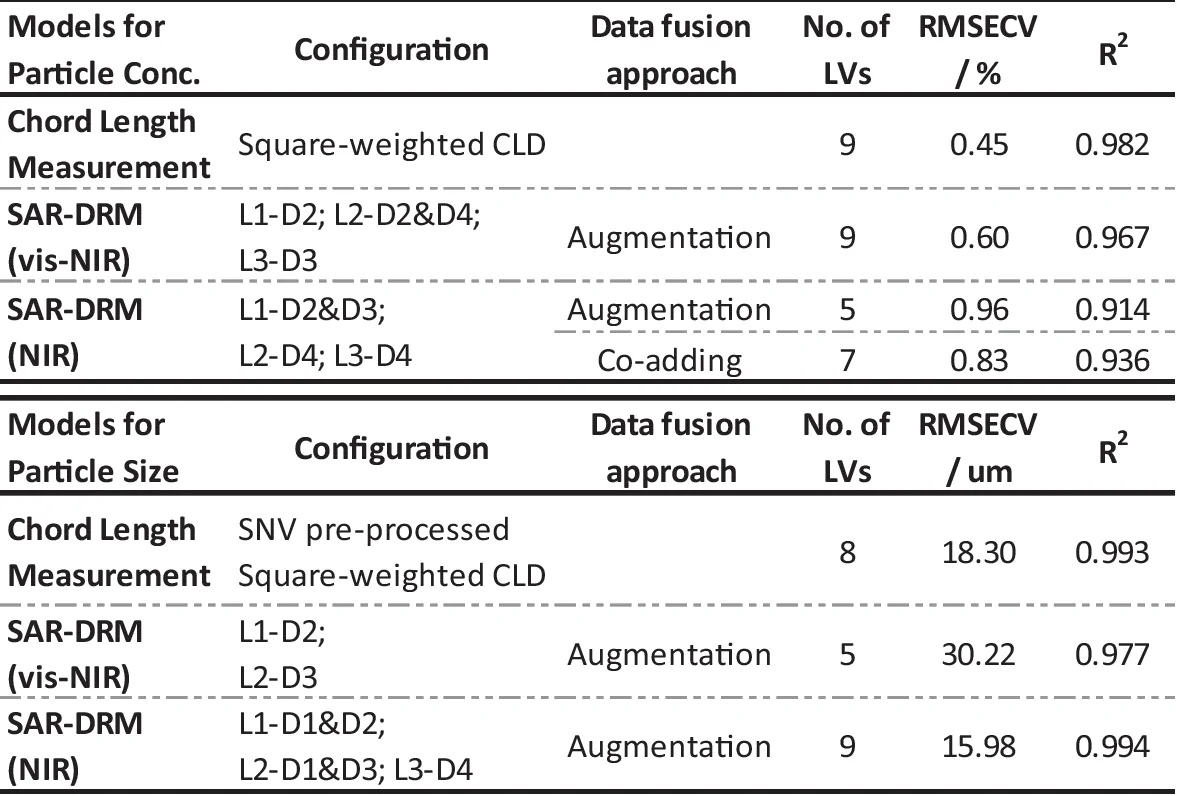
Summary of best-performing models and the corresponding configurations for each measurement technique (and spectral region)

Compared to Table 3, Table 4 indicates that notable improvements in model performance can be achieved by selectively combining measurement configurations for all spectral regions. An increase of over 50% in model performance is observed when selectively combining vis-NIR spectra, and between 25 and 50% for the NIR spectra. The models built on these selectively combined datasets also outperform those using a single source-detector configuration. The best models from the SAR-DRM spectra can reach performance levels comparable to those built on CLDs, with the RMSECV for estimating particle size and concentration reduced to 15.98 µm and 0.6%, respectively. Using the multiple configurations offered by the SAR-DRM system, the systematic analysis highlights the importance and impact of measurement configuration in deriving meaningful spectral information for regression analysis—an aspect currently lacking sufficient focus in instrument development and reflectance measurement design.

### Effect of using one or multiple in-line analytical techniques in estimating particle size and concentration

The comparable performance of SAR-DRM and CLD measurements, as shown in Table 4, is an intriguing discovery. Since the accuracy of CLD measurement is known to be limited by increasing particle concentration, whereas SAR-DRM, conversely, performs better with samples that have higher solid content due to increased reflectance, it would be advantageous to develop a method that combines the strengths of both SAR-DRM and CLD measurements for applications requiring a wide range of particle sizes and concentrations.

One approach to leveraging and integrating the acquired information is to apply the multisensory data fusion method. Data fusion can be conducted at various stages of the overall data analysis process, ranging from combining individual sensor data to decision fusion. Given the complementary nature of the captured information, data fusion should be applied to integrate the SAR-DRM and CLD measurements at the individual data level. In this study, we employ data augmentation to fuse CLDs and spectral data, as the co-adding approach is not suitable for combining measurements with fundamentally different information. Based on the results in Table 4, the combined SAR-DRM configurations are selected for fusion with the CLD data. The same PLSR analysis is performed to evaluate the fused datasets and compare them with the results obtained from the individual measurement techniques. Table 5 summarizes the performance of the PLSR model for the fused CLDs and SAR-DRM vis-NIR and NIR datasets. No pre-processing methods were applied to the fused datasets, as this step has been evaluated in the analysis of individual techniques.

**Table 5:**
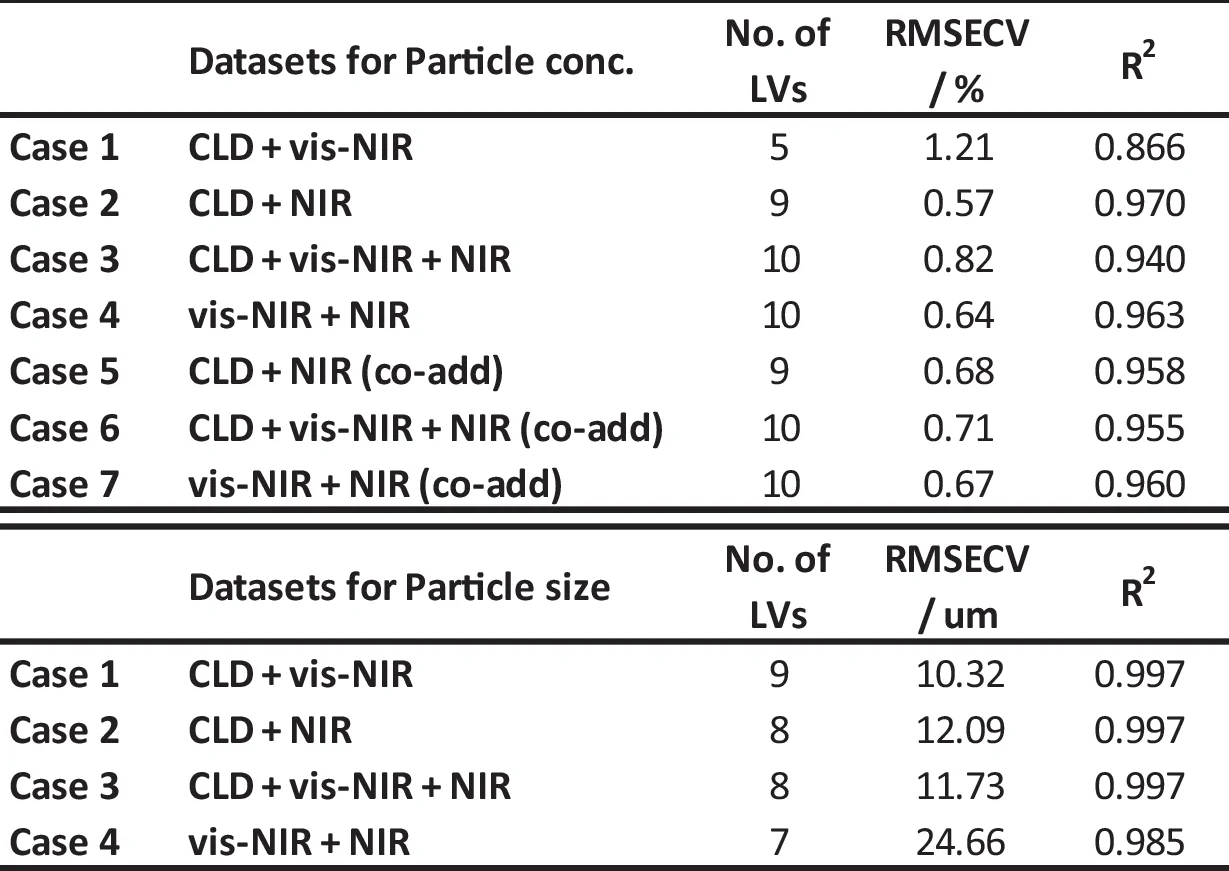
Summary of model performance for the datasets fused using those selected in Table 4

For estimating particle concentration, similar results are seen when the NIR dataset is combined with another technique. Both the augmented and co-added NIR datasets listed in Table 4 are used for data fusion, with the results shown in Cases 2–4 and 5–7, respectively, in Table 5. Overall, fusing NIR data with either CLD or vis-NIR improves the RMSECV of the model compared to models built solely on NIR data. However, combining CLD and vis-NIR appears to negatively affect the models, as seen in Cases 1, 3, and 6. Although merging multiple sensing techniques does not further improve the model’s estimation of particle concentration, a notable insight can be drawn from comparing Cases 2–4 and 5–7 in Table 5.

**Fig. 6:**
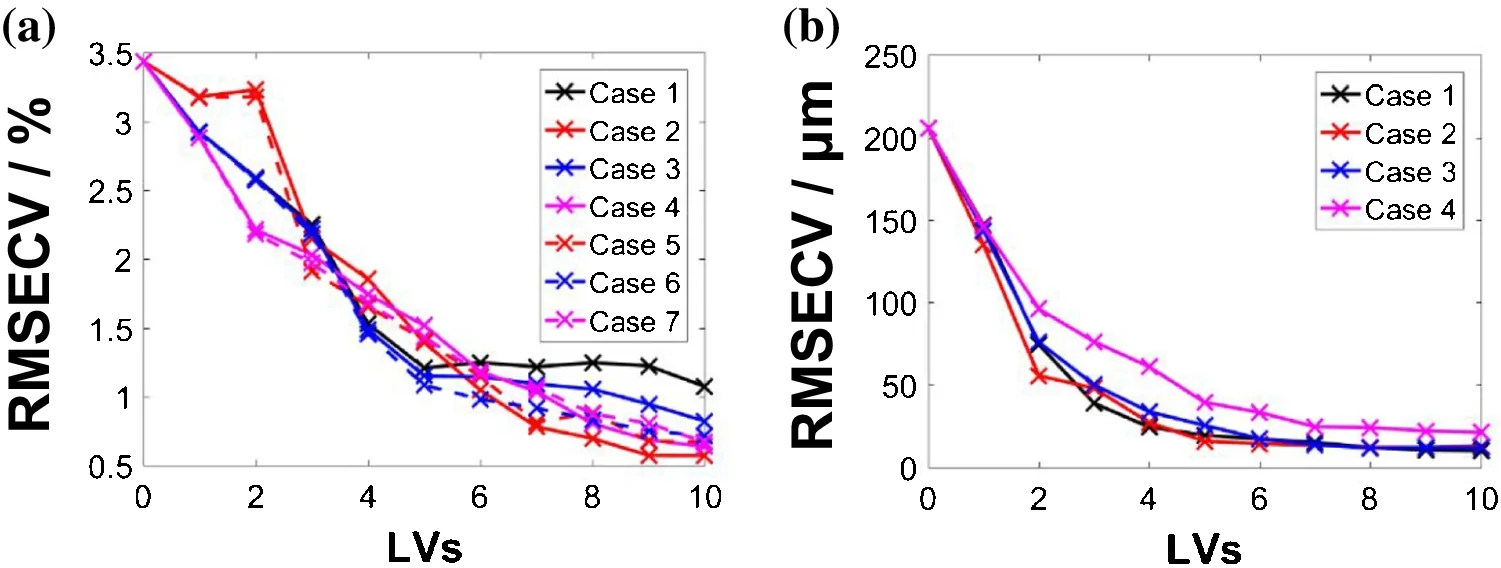
RMSECV curves for PLSR model on **a** particle concentration and **b** particle diameter in Table

Since the augmentation approach yields more variables in the fused dataset than the co-adding approach, one may question whether and how these additional variables, along with the proportion of information in the datasets, influence the calibration model. The CLDs and SAR-DRM spectra of each fiber configuration provide 350 variables (in terms of chord length and wavelength). However, augmenting NIR spectra with four fiber configurations produces a dataset of 1400 variables, whereas the co-adding approach yields the same 350 variables. In the dataset for Case 2, the variables from the CLD comprise only 20% of the total variables. This proportion increases to 50% for Case 5. Therefore, it could suggest that the CLDs have a greater influence on the PLSR model for Case 5 due to the increased contribution of the variables used in modelling. Yet, the comparison of Cases 2–4 and 5–7 in Table 5 indicates that the PLSR analysis models the information contained within the data, not the length of the data. Similar RMSECV curves are observed in Fig. 6a for models built using the same measurement techniques, such as between Cases 2 and 5, which results in choosing the best model with the same number of LVs and achieving a comparable RMSECV. This is also supported by the prediction and residual plots shown in SFigure 8 in the Electronic Supplementary Material.

Although it is common in data fusion to assign weights to information from different sources, this study indicates that the complementary nature of the techniques used could play a more vital role in enhancing model performance. The differentiable information provided by each technique, rather than the dataset size, has a greater impact on the PLSR model and can be used in the modelling. The findings here are consistent with a previous study, which suggests that the PLSR analysis uses only differentiable information to model the data [7].

**Fig. 7:**
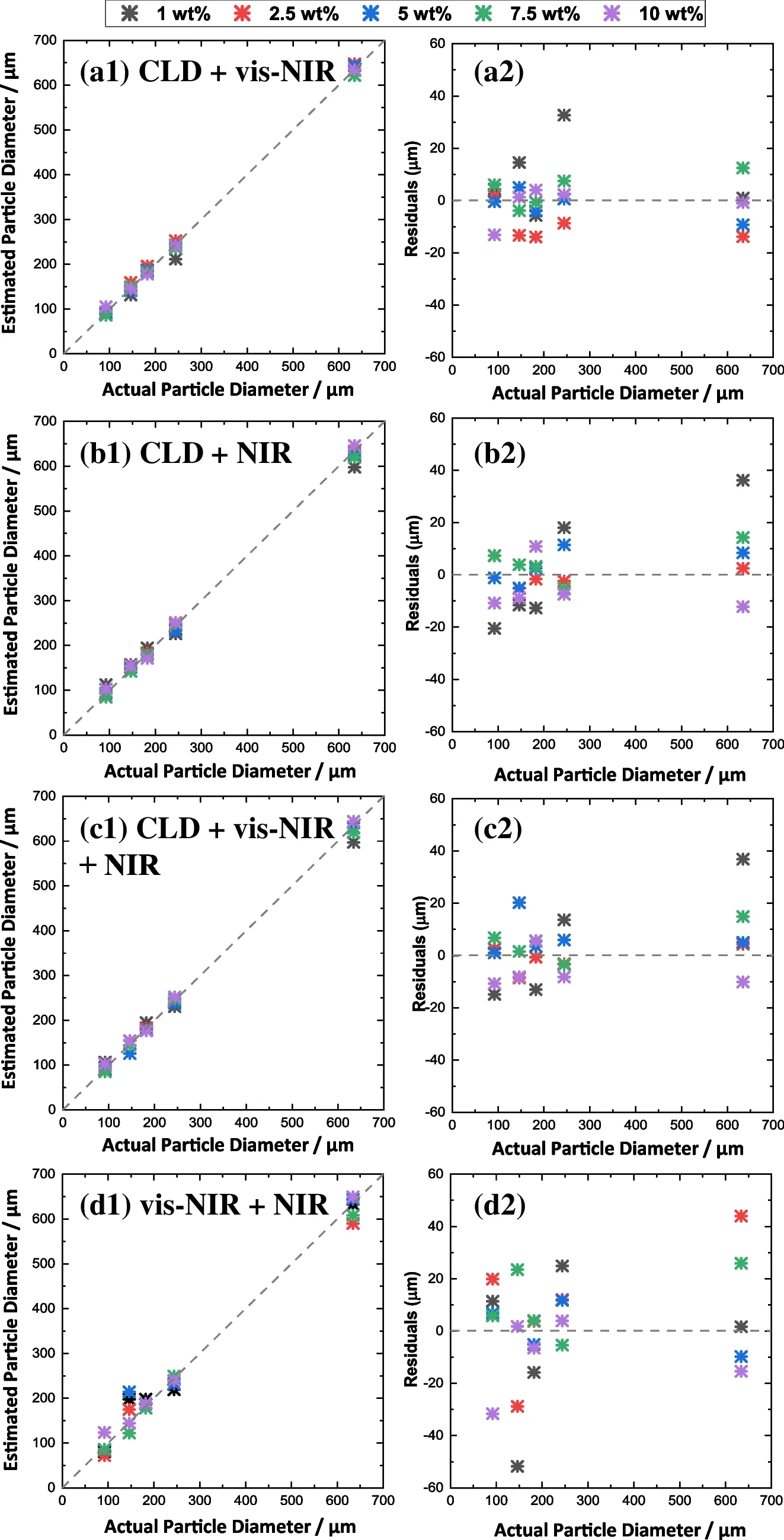
**a1**–**d1** Prediction and **a2**–**d2** residuals of particle diameter estimation using the fused dataset. Plots a–d correspond to Cases 1–4 of the particle size models in Table 5

For estimating particle size, the fused multisensory dataset shows a significant improvement in the PLSR model performance, with the RMSECV lowered to 10–12 µm when the CLD data is included. Comparing the fused models with those from individual techniques reveals a noticeable reduction in errors for almost all particle size groups, except PS630-800, as shown in Fig. 7. The improvement across most particle size groups can be attributed to the PLSR models, which leverage complementary information within the dataset. For instance, incorporating the more pronounced NIR spectral differences for PS25-180, PS180-255, and PS255-350, along with the subtle differences in CLDs, could provide distinct information to improve the model. The model could also utilize the improved data quality from the smoother CLDs to reduce estimation errors. Consequently, similar levels of accuracy are observed across the four smaller particle size groups for datasets containing the CLDs and SAR-DRM spectra.

The observation of similar errors in PS630-800 from the augmented dataset, compared to those from individual techniques, can be explained by PS630-800 being the least preferred sample group for both SAR-DRM and CLD measurements. As shown in Fig. 4, the particle number density for PS630-800 is at least an order of magnitude lower than that of the other particle groups. This results in low turbidity for the PS630-800 samples, leading to weak intensity and a lower signal-to-noise ratio for reflectance measurements such as SAR-DRM, as demonstrated in Fig. 1 and SFigure 3 in the Electronic Supplementary Material. Conversely, while low turbidity benefits CLD measurements, previous studies also indicate that the undesired chord-splitting effect poses significant challenges when analyzing CLDs of large transparent particles such as PS630-800 [8]. Chord splitting leads to higher counts at shorter chord lengths, which can mislead the model when using CLDs from smaller particle sizes and increase estimation errors.

Another interesting observation from Table 5 is that the augmented vis-NIR and NIR data do not improve model performance beyond the individual methods alone. Fusing the vis-NIR and NIR datasets (Case 4) only results in an RMSECV similar to the mean of the RMSECV for the separate spectral regions (16 and 30 µm). This could be due to the particle size effect manifesting similarly in the variation of the vis-NIR and NIR spectral baseline, as seen in Fig. 1 and SFigure 3. This may indicate minimal improvement in the information contrast of the augmented dataset, thus leading to the observed outcomes for Case 4.

Although estimating PSD from SAR-DRM still requires further investigation, the technique shows promise for measuring high-turbidity samples, complementing current in-line methods such as imaging and CLD analysis. The analysis in Table 5 also explores key questions related to fusing SAR-DRM spectra with other techniques, including the source of model improvements and considerations regarding data size and quality. This study demonstrates, for the first time, the capability of SAR-DRM to analyze micron-sized particle suspensions and shows how its complementary information can improve accuracy. It would be worth noting that this study is limited to the sample choice investigated. For applying SAR-DRM in pharmaceutical manufacturing, further understanding of other effects such as particle morphologies, optical interference such as spectral signature, fluorescence, and absorption will need to be achieved. Nevertheless, this study presents encouraging results to support further investigation and development for SAR-DRM to enable more reliable in-line analysis of turbid systems. The results from the multi-sensor analysis also illustrate an alternative approach to obtain particle information without inverting particle measurement using a complex physical model.

## Conclusions

This study demonstrates, for the first time, the potential of SAR-DRM for in-line analysis of micrometer-sized particle suspensions. By utilizing multi-wavelength reflectance data across various spatial and angular configurations, SAR-DRM was shown to be highly sensitive to changes in both particle size and concentration. The spectral responses revealed systematic trends with different particle properties and measurement geometries, which were effectively captured and modelled using partial least squares regression (PLSR). Notably, combining select SAR-DRM configurations through data augmentation significantly enhanced model accuracy while reducing complexity compared to traditional co-adding methods. Furthermore, integrating SAR-DRM data with chord length distribution (CLD) measurements yielded improved models, especially for estimating particle size, by leveraging the complementary strengths of the two techniques. The hybrid models achieved RMSECV values of 10–12 µm, surpassing those of single-sensor models and underscoring the potential of multisensory data fusion in complex particulate systems. While some limitations remain for larger, low-density particles, the findings clearly demonstrate the added value of SAR-DRM in high-turbidity conditions, where conventional in-line methods often struggle. Overall, SAR-DRM presents a novel and powerful approach for real-time particle monitoring, with the flexibility to complement existing process analytical technologies (PAT). Its unique sensitivity to scattering behavior across spatial, angular, and spectral dimensions makes it an excellent candidate for future implementation in pharmaceutical manufacturing and other applications involving complex suspensions or crystallization processes.

## Supplementary Information

Below is the link to the electronic supplementary material.

Supplementary file1 (DOCX 2.34 MB)

## Data Availability

All data underpinning this publication are openly available from the University of Strathclyde at https://doi.org/10.15129/21430cab-0b60-451b-9986-bb2da25375e6.
