# Supplementary material for: Spatially and angularly resolved spectroscopy for in situ estimation of particle size and concentration in particle suspensions
Source: Anal Bioanal Chem. 2026 Jul 29;418(21):7003–17. doi: 10.1007/s00216-026-06696-8 (PMC13658582; doi:10.1007/s00216-026-06696-8)
Supplement: Supplementary file 1 [file 216_2026_6696_MOESM1_ESM.docx]

# Supplementary figures

**SFigure 1: a)** Volume-based particle size distributions and **b)** cumulative size distributions of all particle size groups studied.

**SFigure 2:** a) Image of the experimental setup for SAR-DRM and CLD measurements. b) Spatial and angular arrangements of the source and detecting fibers of the SAR-DRM probe.

**SFigure 3:** Effect of particle diameter on SAR-DRM spectra in the NIR region using a)-c) 0, 30 and 45° incident light and 1)-4) 0.3-1.2 mm spatial distances. 10 wt.% particle concentration is used for all samples

**SFigure 4:** Effect of particle concentration of PS125-180 in NIR region, when the light is emitted at **a)** 0, **b)** 30 and **c)** 45° and collected from the 4 different distances from the light source: **1** (0.3 mm), **2** (0.6 mm), **3** (0.9 mm) and **4** (1.2 mm). Particle diameter group used – PS125-180.

**STable 1:** Summary of PLSR models for all available vis-NIR and NIR measurement configurations investigated in Steps 1 using D10 and D90 of the particle size distribution as the dependent variables.

**RMSECV / %**

**RMSECV / µm**

**SFigure 5:** RMSECV curves for models built using datasets in Table 4 for estimating particle diameter and concentration, (a) and (b), respectively.

**SFigure 6:** (a1)-(d1) prediction and (a2)-(d2) residuals of particle concentration estimation from models using individual techniques. Plots (a)-(d) correspond to models built on CLD, augmented vis-NIR and NIR, and co-added NIR data, respectively.

**SFigure 7:** (a1)-(c1) prediction and (a2)-(c2) residuals of particle concentration estimation from models using individual techniques. Plots (a)-(c) correspond to models built on CLD and augmented vis-NIR and NIR spectra, respectively.

**SFigure 8:** (a1)-(g1) prediction and (a2)-(g2) residuals of particle concentration estimation using fused dataset. Plots (a)-(g) correspond to Casex 1-7 of the particle concentration models in Table 5.
